# Cardiac morphology and flow dynamics in children born to hypertensive pregnancies

**DOI:** 10.1093/ehjimp/qyag145

**Published:** 2026-08-20

**Authors:** Prenali Dwisthi Sattwika, Katie Suriano, João Filipe Fernandes, Tamara den Harink, Christina Yi Ling Aye, Yvonne Kenworthy, Annabelle McCourt, Betty Raman, Zakariye Ashkir, Aaron Timothy Hess, Joana Leal Pelado, Federica Viola, Tino Ebbers, Carl-Johan Carlhäll, Petter Dyverfeldt, Adelaide de Vecchi, Winok Lapidaire, Paul Leeson, Pablo Lamata, Adam James Lewandowski

**Affiliations:** Division of Cardiovascular Medicine, Radcliffe Department of Medicine, Cardiovascular Clinical Research Facility, University of Oxford, Level 1, Oxford Heart Centre, John Radcliffe Hospital, Headley Way, Headington, Oxford, UK; Department of Internal Medicine, Faculty of Medicine, Public Health, and Nursing, Universitas Gadjah Mada, Yogyakarta, Indonesia; Division of Cardiovascular Medicine, Radcliffe Department of Medicine, Cardiovascular Clinical Research Facility, University of Oxford, Level 1, Oxford Heart Centre, John Radcliffe Hospital, Headley Way, Headington, Oxford, UK; Department of Digital Twins for Healthcare, School of Biomedical Engineering and Imaging Sciences, King's College London, London, UK; Department of Epidemiology and Data Science, Amsterdam University Medical Center, University of Amsterdam, Amsterdam, The Netherlands; Division of Cardiovascular Medicine, Radcliffe Department of Medicine, Cardiovascular Clinical Research Facility, University of Oxford, Level 1, Oxford Heart Centre, John Radcliffe Hospital, Headley Way, Headington, Oxford, UK; Nuffield Department of Women's and Reproductive Health, University of Oxford, Oxford, UK; Oxford Fetal Medicine Unit, John Radcliffe Hospital, Oxford University Hospitals, Oxford, UK; Division of Cardiovascular Medicine, Radcliffe Department of Medicine, Cardiovascular Clinical Research Facility, University of Oxford, Level 1, Oxford Heart Centre, John Radcliffe Hospital, Headley Way, Headington, Oxford, UK; Division of Cardiovascular Medicine, Radcliffe Department of Medicine, Cardiovascular Clinical Research Facility, University of Oxford, Level 1, Oxford Heart Centre, John Radcliffe Hospital, Headley Way, Headington, Oxford, UK; Division of Cardiovascular Medicine, Radcliffe Department of Medicine, Oxford Centre for Clinical Magnetic Resonance Research, University of Oxford, Oxford, UK; Division of Cardiovascular Medicine, Radcliffe Department of Medicine, Oxford Centre for Clinical Magnetic Resonance Research, University of Oxford, Oxford, UK; Nuffield Department of Clinical Neurosciences, Centre for Integrative Neuroimaging, FMRIB, University of Oxford, Oxford, UK; Division of Cardiovascular Medicine, Radcliffe Department of Medicine, Oxford Centre for Clinical Magnetic Resonance Research, University of Oxford, Oxford, UK; Department of Medical and Health Sciences, Unit of Cardiovascular Sciences, Linköping University, Linköping, Sweden; Department of Medical and Health Sciences, Unit of Cardiovascular Sciences, Linköping University, Linköping, Sweden; Center for Medical Image Science and Visualization, Linköping University, Linköping, Sweden; Science for Life Laboratory, Linköping University, Linköping, Sweden; Department of Medical and Health Sciences, Unit of Cardiovascular Sciences, Linköping University, Linköping, Sweden; Department of Clinical Physiology in Linköping, Region Östergötland, Linköping, Sweden; Department of Medical and Health Sciences, Unit of Cardiovascular Sciences, Linköping University, Linköping, Sweden; Center for Medical Image Science and Visualization, Linköping University, Linköping, Sweden; Science for Life Laboratory, Linköping University, Linköping, Sweden; Department of Digital Twins for Healthcare, School of Biomedical Engineering and Imaging Sciences, King's College London, London, UK; Division of Cardiovascular Medicine, Radcliffe Department of Medicine, Cardiovascular Clinical Research Facility, University of Oxford, Level 1, Oxford Heart Centre, John Radcliffe Hospital, Headley Way, Headington, Oxford, UK; Division of Cardiovascular Medicine, Radcliffe Department of Medicine, Cardiovascular Clinical Research Facility, University of Oxford, Level 1, Oxford Heart Centre, John Radcliffe Hospital, Headley Way, Headington, Oxford, UK; Department of Digital Twins for Healthcare, School of Biomedical Engineering and Imaging Sciences, King's College London, London, UK; Division of Cardiovascular Medicine, Radcliffe Department of Medicine, Cardiovascular Clinical Research Facility, University of Oxford, Level 1, Oxford Heart Centre, John Radcliffe Hospital, Headley Way, Headington, Oxford, UK; Nuffield Department of Population Health, University of Oxford Big Data Institute, Old Road Campus, Oxford, OX3 7LF, UK

**Keywords:** hypertensive pregnancies, paediatrics, cardiovascular magnetic resonance imaging, cardiac shape, four-dimensional flow, kinetic energy

## Abstract

**Aims:**

Hypertensive pregnancies affect up to 10% of pregnancies globally and are linked to greater cardiovascular risk in offspring. Subclinical cardiac changes have been reported across developmental stages. This study aimed to assess whether children born to hypertensive pregnancies exhibit altered left ventricular (LV) morphology and cardiac flow characteristics.

**Methods and results:**

In this cross-sectional study, cardiovascular magnetic resonance (CMR) was performed on a 3.0-Tesla Siemens PRISMA scanner in children aged 6–9 years: 44 offspring of hypertensive pregnancies (50% female) and 56 controls from normotensive pregnancies (37.5% female). Free-breathing real-time cine images were acquired to assess structure and function and to create a computational LV shape atlas. Four-dimensional (4D) Flow CMR was acquired to quantify LV flow component volumes and kinetic energy (KE), enabling assessment of intraventricular flow organization and energetics. Children born to hypertensive and normotensive pregnancies were matched in age (7.49 ± 0.73 and 7.41 ± 1.12 years), but those from hypertensive pregnancies had significantly shorter gestations (median 37.3 weeks [IQR 35.6–39.0] vs. 39.0 weeks [37.7–40.8], *P* = 0.002). Conventional cardiac measures did not differ between groups. Exploratory shape analysis identified subtle group-level LV differences, including relative prominence of the basal anterior wall, and a more convex LV outflow tract (*P* < 0.01; AUC = 0.654). LV retained inflow end-diastolic KE was lower in exposed offspring (3.35 [3.03–4.80] vs. 5.70 [4.35–6.80] μJ/mL; *P* < 0.001).

**Conclusion:**

Children born to hypertensive pregnancies show subtle group-level differences in LV morphology and diastolic flow energetics. These findings suggest early differences in cardiovascular phenotype, although their long-term clinical significance remains uncertain and requires longitudinal investigation.

**Registration:**

URL: https://www.clinicaltrials.gov; Unique identifiers: NCT05698836.

**Lay Summary:**

This study investigated whether children born to mothers with high blood pressure during pregnancy show early differences in heart structure and blood flow.

- Children exposed to high blood pressure during pregnancy had subtle differences in heart shape and blood flow patterns.
- The differences identified were small and could only be detected using advanced heart imaging analyses. They were seen when comparing groups of children rather than being useful for identifying individual children, and their long-term importance is not yet known.

## Graphical Abstract

**Graphical Abstract:**
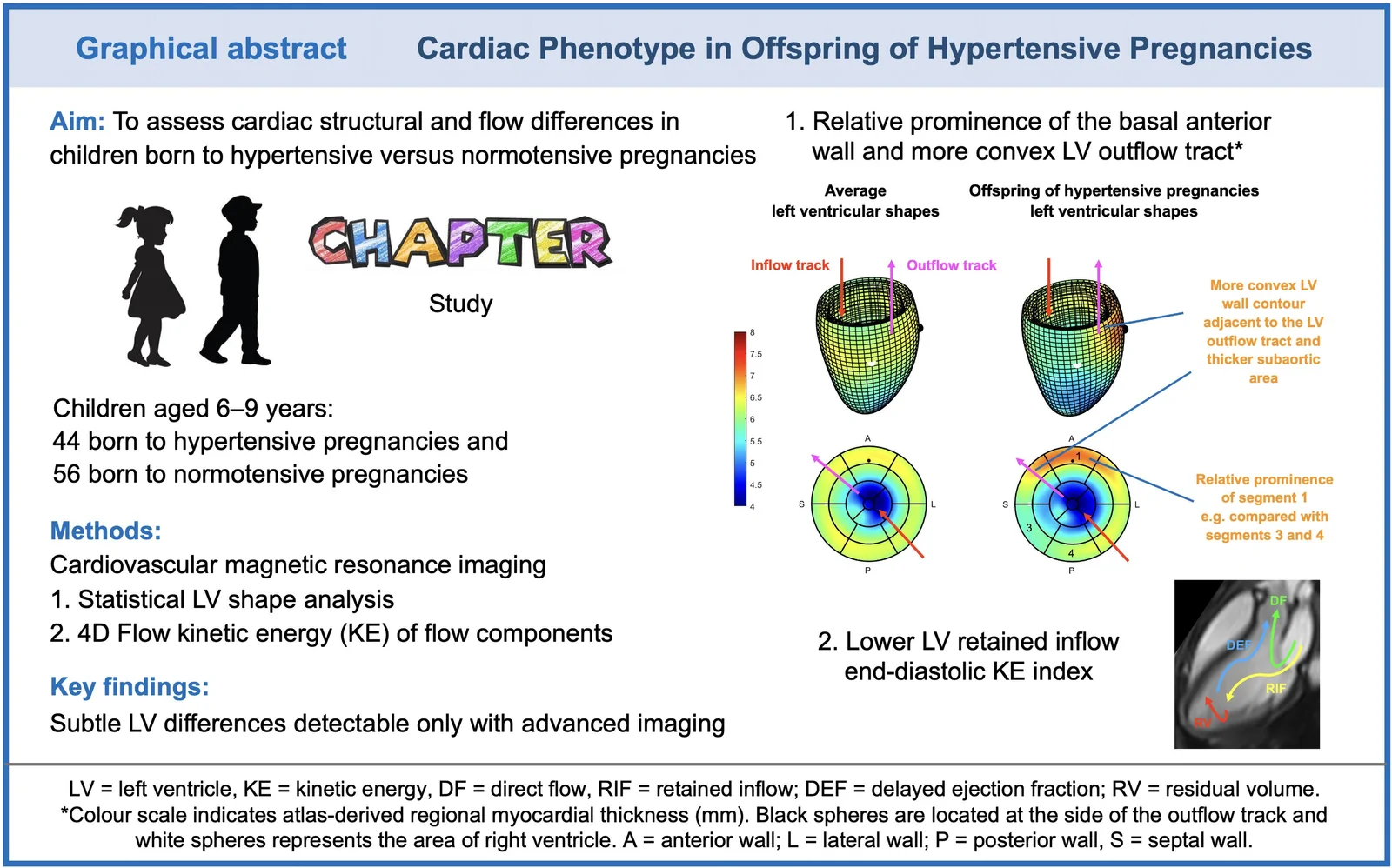

## Introduction

Hypertensive pregnancies affect ∼10% of pregnancies^1^ and are associated with increased cardiovascular risk in offspring.^2^ These risks likely reflect interactions between genetic susceptibility, altered foetal programming, and postnatal environmental factors.^3^ Epidemiological studies report a higher incidence of myocardial infarction and stroke in offspring of hypertensive pregnancies, with population-based data showing hazard ratios of 1.33 for ischaemic heart disease and 1.34 for stroke compared with unexposed offspring.^4,5^ Whether this increased cardiovascular risk is partly attributable to early subclinical cardiac remodelling remains unclear.

Cardiovascular studies across developmental stages have reported alterations in left ventricular (LV) and right ventricular (RV) structure and function in offspring of hypertensive pregnancies.^6–8^ However, evidence remains mixed, with modest and inconsistent alterations reported.^9^ No single conventional cardiac imaging metric has consistently identified structural or functional remodelling in childhood.^9^ More advanced cardiovascular magnetic resonance (CMR) approaches, including computational shape modelling and four-dimensional (4D) Flow CMR imaging, may identify early anatomical and functional changes.^10,11^ These tools enable assessment of ventricular geometry and intraventricular blood flow energetics,^12,13^ potentially revealing cardiovascular adaptations not captured by conventional CMR measures.

We aimed to determine whether children born to hypertensive pregnancies exhibit early alterations in cardiac structure and flow characteristics. We used CMR to assess LV and RV morphology, statistical shape analysis to characterize LV shape, and 4D Flow CMR to quantify intracardiac kinetic energy (KE) of blood flow components. These measures were compared between children born to hypertensive vs. normotensive pregnancies. We hypothesized that exposed offspring would demonstrate differences in LV morphology and intraventricular flow energetics.

## Methods

### Study design and subjects

The CHAPTER study (Cardiovascular Health Assessment of Preterm and TERm-born children) is a prospective cohort study of 155 children with multiple clinic assessments between the ages of 3 and 12 years, including a CMR scan at age 6–9 years. This cross-sectional CMR study was conducted as a pre-specified sub-study within the CHAPTER cohort, using a standardized imaging protocol.^14^ The CHAPTER study was approved by the West Midlands—The Black Country Research Ethics Committee (Reference 18/WM/0131) and registered on ClinicalTrials.gov (NCT05698836).

Children and their parents or legal guardians attended study visits at the Oxford Cardiovascular Clinical Research Facility and the University of Oxford Centre for Clinical Magnetic Resonance Research. Written informed consent was obtained from parent/legal guardian, with child assent where applicable. This study was conducted in accordance with the principles of the Declaration of Helsinki and Good Clinical Practice.

The inclusion criteria were as follows: male or female, previously took part in the infancy study performed by our research group (the EPOCH study, Effect of Prematurity and hypertensive disorders of pregnancy on Offspring Cardiovascular Health)^6^ or the participant’s friend or sibling took part with available birth history information, and aged between 3 and 9 years at the time of enrolment. The exclusion criteria were as follows: evidence of congenital heart disease, significant chronic disease relevant to cardiovascular or metabolic status, and for the CMR study between ages 6 and 9 years, any contraindications to CMR, or unsuitability for CMR based on the responses to the CMR safety screening form.

### Identification of exposures

Maternal hypertensive status, gestational age, and birthweight were ascertained from medical records. Additional prospective data on medical history, lifestyle, and family history were collected via a parent-reported questionnaire. Hypertensive disorders of pregnancy were classified using established definitions derived from maternal records, in accordance with the 2018 International Society for the Study of Hypertension in Pregnancy criteria.^6,15^ Hypertension was defined as systolic blood pressure (SBP) ≥140 mmHg and/or diastolic blood pressure (DBP) ≥90 mmHg arising after 20 weeks’ gestation. Gestational hypertension was defined as new-onset hypertension without proteinuria or features of preeclampsia. Preeclampsia was defined as new-onset hypertension with proteinuria and/or evidence of maternal organ dysfunction and/or uteroplacental dysfunction (including foetal growth restriction).

### CMR study visit measures

#### Anthropometrics and blood pressure

Height, weight, and blood pressure measurements were collected by trained clinical research professionals. Height was measured to the nearest 0.1 cm and weight to the nearest 0.1 kg with participants wearing light clothing. After a 5-min rest, three brachial blood pressure readings were taken on the left arm at 1-min intervals using the CARESCAPE™ Dinamap V100 (GE Healthcare, UK). The final two readings were averaged when available.

#### Physical activity measurements

Participants were instructed to wear a GENEActiv accelerometer for 7 days to assess physical activity levels. This wrist-worn device objectively measured sedentary, light, and moderate-to-vigorous physical activity. Data were analysed using tools available at https://github.com/OxWearables/biobankAccelerometerAnalysis.

#### Cardiac magnetic resonance image acquisition

Cardiac structure and function were investigated using a 3.0-Tesla CMR scanner with an 18-channel cardiac surface coil (3T PRISMA, Siemens, Munich, Germany). Real-time steady-state free-precession cine images with retrospective gating and shortened free breathing were acquired to obtain localization images, followed by optimized horizontal long axis, vertical long axis, LV outflow tract (LVOT), and short-axis views. A short-axis stack covering the entire LV length was obtained with a 3 mm inter-slice gap and a slice thickness of 7 mm.

4D Flow CMR was acquired using a free-breathing, retrospective ECG-triggered, and respiratory-gated sequence. Standard acquisition parameters included a velocity encoding of 120 cm/s, repetition time of 4.9 ms, echo time of 2.65 ms, and flip angle of 7°. The temporal resolution was 38.88 ms, with an acquired spatial resolution of 2.2 mm isotropic voxels.

#### Cardiac conventional structure and function analysis

CMR images were analysed using cvi42 software (version 5.13.7, Circle Cardiovascular Imaging Inc., Calgary, Alberta, Canada) following established methods.^16,17^ Ventricular volumes and function were derived by contouring endocardial borders at end-diastole and end-systole. Epicardial borders were contoured at end-diastole to assess LV mass, calculated using a myocardial density of 1.05 g/cm^3^. LV and RV end-diastolic and end-systolic volumes were used to derive stroke volume, and ejection fraction was calculated as stroke volume/end-diastolic volume. Ventricular volumes, stroke volume, and LV mass were indexed to height raised to the power of 2.7 (m^2.7^).

LV wall thickness and cavity diameter were measured on the basal short-axis slice at end-diastole. Relative wall thickness was calculated as twice the inferolateral wall thickness divided by LV internal diameter. Ventricular length was measured from the basal plane to the apex in the horizontal long-axis view. Myocardial deformation was assessed using strain analysis as previously described.^18^

Image analysis was performed in a dedicated core laboratory by a trained reader blinded to group allocation (P.D.S.), with review by senior investigators (A.J.L. and B.R.) where required.

#### Cardiac computational atlas

A computational LV atlas was constructed using a published method.^17,19^ LV endocardial and epicardial borders were segmented at end-diastole and reconstructed, with RV position manually identified. The dataset was converted into binary segmentation images, and high-order meshes were fitted using image registration and variational mesh techniques.

Principal component analysis (PCA) was used to identify key modes of shape variation, reducing dimensionality. Because PCA generates multiple orthogonal modes of shape variation, no individual PCA mode was pre-specified *a priori*. Mode selection was therefore data-driven, and the findings were considered exploratory. Linear discriminant analysis was applied to identify combinations of PCA modes that best distinguished offspring of hypertensive and normotensive pregnancies, as previously described.^10^ The computational atlas methodology has previously been developed, validated, and applied across both paediatric and adult cardiovascular cohorts.^10,17^

#### Cardiac 4D flow CMR analysis for the left ventricle

Four-dimensional flow CMR data were analysed using validated methods.^11,20^ Quality control excluded datasets with stroke volume <80% of conventional measures, ≥15% inflow–outflow discrepancy, or ≥25% non-physiological flow (see Supplementary data online, *Table S1*). LV segmentation was performed at end-diastole and end-systole using Segment software (version 4.0 R12067b, Medviso AB, Lund, Sweden). The segmentation at end-diastole was then resampled to generate a volume with isotropic voxels matching the resolution of the flow data. Pathlines were emitted from the centre of each voxel within the ventricular segmentation and tracked both backwards and forwards in time until the preceding or subsequent end-systole, representing the entire ventricular end-diastolic volume over a complete cardiac cycle. Assessments of left intraventricular flow were performed by P.D.S., who was blinded to exposure group allocation, using an in-house 4D Flow CMR tool developed by the Cardiovascular Imaging and Modelling cluster from Linköping University, Sweden, using previously published methods.^21^

Intraventricular flow was classified into four flow components (direct flow: blood entering and leaving the ventricle within one cardiac cycle; retained inflow: blood entering during diastole but not ejected in the subsequent systole; delayed ejection flow: blood ejected during systole after entering in a prior cycle; and residual volume: blood persisting within the ventricle for at least two cardiac cycles) based on the relative positions of all pathlines within the cardiac chamber, as defined by the end-systolic segmentation.^20^ Each pathline represented a blood volume with known mass (density 1060 kg/m^3^) and velocity. KE was calculated as one-half the product of blood density, pathline volume, and the square of velocity at each time point. KE was calculated for each flow component and indexed to the corresponding flow-component volume (μJ/mL).^11,22^ No additional indexing to LV volume or body size was performed, as this approach provides a standardized measure of flow energetics and facilitates comparison between individuals. Peak systole and peak diastole were defined as the cardiac frames corresponding to the maximum values on the time-resolved KE curve during systole and diastole, respectively.^23^ End-diastole was defined as the final diastolic frame immediately preceding ventricular systole. The 4D Flow analysis pipeline has previously been validated and applied across multiple cardiovascular cohorts using established methodologies.^11,20,21^

### Statistical analysis

Study size was determined by the number of participants with analysable conventional and 4D Flow CMR data. Missing data were handled using complete-case analyses for each outcome. Potential selection bias was assessed by comparing baseline characteristics of included and excluded participants.

Statistical analyses were performed using R (version 4.5.2) and RStudio (version 2025.05.1 + 513). Normality was assessed using visual inspection and the Shapiro-Wilk test. Continuous variables are presented as mean (SD) or median (IQR), as appropriate. Group comparisons were performed using independent *t*-tests or Mann–Whitney *U*-tests. Categorical variables were compared using χ^2^ or Fisher’s exact tests. Statistical significance was set at *P* < 0.05.

Linear regression analyses were performed to assess associations between hypertensive pregnancy exposure and cardiac parameters. Unstandardized regression coefficients (*B*) with 95% CI were reported. Primary models were adjusted for offspring sex and body mass index (BMI). Sequential models additionally included adjustment for gestational age, followed by maternal pre-pregnancy BMI. Maternal BMI was included as a covariate based on previous studies demonstrating associations with offspring cardiac measures.^24^ Sensitivity analysis was conducted in a subset of term-born children (≥37 weeks) to evaluate whether the findings were influenced by prematurity.

Conventional CMR, computational shape modelling, and 4D Flow CMR were pre-specified components of the imaging protocol. Conventional CMR parameters were assessed as predefined measures of cardiac structure and function. In contrast, no individual PCA mode from the statistical shape analysis or individual KE metric from the 4D Flow analyses was pre-specified as a primary endpoint. Accordingly, analyses of individual PCA modes and 4D Flow KE metrics were treated as exploratory, with the findings considered hypothesis-generating.

For 4D Flow CMR analyses, KE was assessed for four LV flow components (direct flow, retained inflow, delayed ejection flow, and residual volume) at three predefined cardiac phases (peak systole, peak diastole, and end-diastole). Between-group comparisons were performed for each cardiac phase, with Bonferroni correction applied across the four flow components (*α* = 0.0125).^22^ This approach was chosen because comparisons within each cardiac phase were considered the primary family of related tests, while preserving interpretability of the temporal changes in KE.

## Results

### Participant baseline characteristics

**Figure 1:**
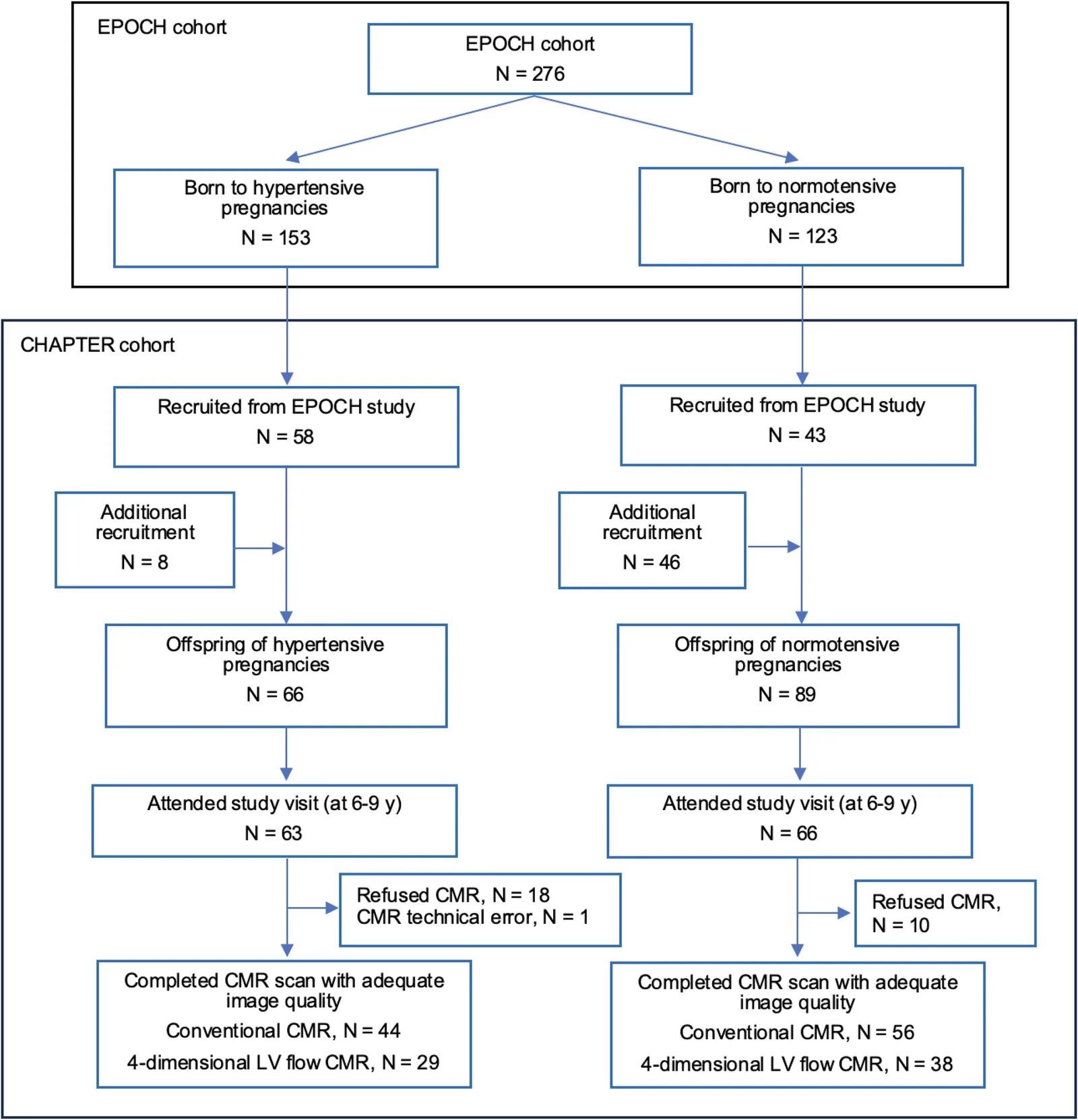
Study flow diagram. Flow diagram of participant recruitment and inclusion in the analytic cohort. EPOCH = Effect of Prematurity and hypertensive disorders of pregnancy on Offspring Cardiovascular Health; CHAPTER = Cardiovascular Health Assessment of Preterm and TERm-born children.

Between April 2019 and August 2023, 155 children were enrolled in the CHAPTER study (*Figure 1*). Of these, 129 attended the study visit at ages 6–9 years, comprising 63 offspring of hypertensive pregnancies and 66 offspring of normotensive pregnancies. CMR scans were completed in 44 offspring of hypertensive pregnancies (50% female) and 56 offspring of normotensive pregnancies (37.5% female). According to the ISSHP classification, 31 offspring (70.5%) were born to pregnancies complicated by preeclampsia (PET) and 13 (29.5%) to pregnancies complicated by gestational hypertension (GH). No offspring were born to mothers with chronic hypertension.

Of the 100 participants with analysable conventional CMR, 95 scans were suitable for computational shape analysis after exclusion of five datasets because of technical limitations. A total of 93 children underwent 4D Flow CMR acquisition (40 offspring of hypertensive pregnancies and 53 controls). Following quality-control assessment, 26 LV datasets were excluded because of image quality or processing constraints, leaving 67 participants (29 offspring of hypertensive pregnancies and 38 controls) for the final analysis. Relative to the conventional CMR cohort, 15/44 (34.1%) offspring of hypertensive pregnancies and 18/56 (32.1%) controls were excluded from the final 4D Flow analysis.

Baseline characteristics are presented in *Table 1*. Offspring of hypertensive pregnancies had lower gestational age and birthweight compared with those born to normotensive pregnancies (mean difference 1.67 weeks, 95% CI 0.35–3.00; *P* = 0.014, and 548 g, 95% CI 195–901; *P* = 0.003, respectively). There were no significant differences in age, BMI, systolic blood pressure, lung capacity, or physical activity levels between groups. No participants had a history of chronic hypertension.

**Table 1:** Descriptive baseline characteristics of participants completed CMR scans

Variables	Hypertensive pregnancies offspring (Overall *N* = 44)	*N*	Normotensive pregnancies offspring (Overall *N* = 56)	*N*	*P-*value
Maternal characteristics					
Maternal age at delivery, years	33.27 (5.23)	44	33.02 (3.7)	47	0.79
Systolic blood pressure, mmHg					
Early pregnancy	120 (110–125)	42	110 (104–118)	47	**<0**.001*
Mid pregnancy	120 (112–130)	41	110 (104–118)	45	**<0**.001*
Late pregnancy	130 (120–138)	41	114 (110–124)	45	**<0**.001*
Highest blood pressure	163 (153–178)	41	124 (120–130)	46	**<0**.001*
Diastolic blood pressure, mmHg					
Early pregnancy	73 (66–80)	42	65 (60–70)	47	**<0**.001*
Mid pregnancy	76 (70–85)	41	65 (60–70)	45	**<0**.001*
Late pregnancy	82 (80–90)	41	70 (60–75)	45	**<0**.001*
Highest blood pressure	101 (98–108)	41	75 (70–83)	46	**<0**.001*
Body mass index at booking, kg/m^2^	25.4 (22.4–29.09)	43	22.0 (21–23.7)	47	**<0**.001*
Maternal hypertension during pregnancy		44		56	NA
Preeclampsia	31 (70.45%)		—		
Gestational hypertension	13 (29.55%)		—		
Gestational diabetes mellitus	4 (9.09%)	44	2 (4.26%)	47	0.42
Smokers	0 (0%)	44	0 (0%)	56	>0.99
Offspring characteristics					
Age, years	7.49 (0.73)	44	7.41 (1.12)	56	0.68
Male	22 (50%)	44	35 (62.5%)	56	0.29
Gestational age, weeks	37.3 (35.6–39)	44	39 (37.7–40.8)	56	**0**.002*
≥ 37 weeks	24 (54.54%)		44 (78.57%)		0.05
32–36 ^+^ ^6^ weeks	16 (36.36%)		9 (16.07%)		
28 to 31 ^+^ ^6^ wk	3 (6.82%)		2 (3.57%)		
< 28 weeks	1 (2.27%)		1 (1.79%)		
Prematurity	20 (45.45%)	44	12 (21.43%)	56	**0**.019*
Antenatal steroids	15 (34.09%)	44	8 (17.02%)	47	0.10
Birth weight, g	2672.5 (2160–3392.5)	44	3365 (2874–3782.5)	56	**0**.002*
Anthropometrics at study visit					
Weight, kg	26.2 (5.2)	44	27.4 (6.6)	56	0.35
Height, cm	125.3 (6.8)	44	127.7 (9.6)	56	0.15
Body mass index, kg/m^2^	16.6 (2.3)	44	16.5 (2.1)	56	0.89
Vital signs at study visit					
Systolic blood pressure, mmHg	105 (8)	42	104 (8)	56	0.46
Diastolic blood pressure, mmHg	59 (5)	42	60 (5)	56	0.68
Heart rate, beats/min	85 (11)	42	83 (12)	56	0.48
Spirometry data					
Forced vital capacity (FVC), L	1.69 (0.47)	42	1.85 (0.47)	54	0.10
Forced expiratory volume in half a second (FEV0.5), L	0.89 (0.30)	42	0.96 (0.31)	54	0.32
Forced expiratory volume in one second (FEV1), L	1.28 (0.33)	42	1.38 (0.35)	54	0.14
FEV1/FVC	0.78 (0.13)	42	0.76 (0.12)	54	0.52
Physical fitness					
Sedentary behaviour, hours/day	8.89 (1.47)	38	8.43 (1.50)	43	0.17
Light physical activity, hours/day	4.49 (1.21)	38	4.71 (1.24)	43	0.43
Moderate to vigorous physical activity, hours/day	1.70 (0.87)	38	1.75 (0.57)	43	0.75

NA = not applicable.

Data presented as mean (standard deviation) for parametric data or median (interquartile range) for non-parametric data or *N* (%). *N* indicates the number of participants with available data for each variable.

*P < 0.05.

### Conventional cardiac structure and function

Standard CMR-derived measures of ventricular structure and function, including volumes, ejection fraction, and strain, did not differ significantly between groups (*Table 2*). Comparisons of offspring born to PET and GH also showed no differences.

**Table 2:** Cardiac structure and function of participants

	Hypertensive pregnancies offspring	*N*	Normotensive pregnancies offspring	*N*	*P*-value
CMR ventricular structural parameters					
LV mass, g	43.24 (6.91)	44	46.19 (10.94)	56	0.10
LV mass index, g/m^2.7^	23.68 (4.10)	44	23.75 (3.85)	56	0.93
LV end-diastolic volume, mL	65.89 (8.32)	44	69.51 (14.43)	56	0.12
LV end-diastolic volume, mL/m^2.7^	36.06 (5.45)	44	35.78 (4.77)	56	0.78
LV mass/end-diastolic volume, g/mL	0.66 (0.09)	44	0.66 (0.07)	56	0.73
LV relative wall thickness	0.27 (0.05)	44	0.28 (0.05)	56	0.43
LV length at end-diastole, mm	70.04 (4.67)	44	70.68 (6.63)	56	0.57
RV end-diastolic volume, mL	69.18 (9.23)	44	72.22 (15.90)	56	0.23
RV end-diastolic volume, mL/m^2.7^	37.85 (5.91)	44	37.09 (4.86)	56	0.48
RV base diameter at end-diastole, mm	35.86 (4.15)	44	36.46 (4.17)	56	0.48
RV mid diameter at end-diastole, mm	25.26 (4.26)	44	25.90 (3.66)	56	0.42
RV length at end-diastole, mm	62.65 (5.32)	44	64.17 (6.59)	56	0.22
CMR ventricular functional parameters					
LV stroke volume, mL	39.37 (5.26)	44	41.21 (8.32)	56	0.18
LV stroke volume, mL/m^2.7^	21.56 (3.44)	44	21.29 (3.20)	56	0.68
LV ejection fraction, %	59.86 (4.30)	44	59.53 (4.37)	56	0.71
LV longitudinal systolic strain, %	−18.40 (2.02)	44	−18.68 (2.06)	56	0.51
LV longitudinal systolic strain rate, s^−1^	−1.05 (0.16)	44	−1.04 (0.15)	56	0.75
LV basal circumferential systolic strain, %	−18.71 (2.38)	44	−18.84 (1.73)	56	0.75
LV basal circumferential systolic strain rate, s^−1^	−1.17 (0.14)	44	−1.12 (0.16)	56	0.08
LV mid circumferential systolic strain, %	−17.97 (2.09)	44	−18.30 (1.96)	56	0.43
LV mid circumferential systolic strain rate, s^−1^	−1.11 (0.12)	44	−1.11 (0.12)	56	0.90
LV apical circumferential systolic strain, %	−20.56 (2.95)	44	−20.19 (3.26)	56	0.56
LV apical circumferential systolic strain rate, s^−1^	−1.44 (0.19)	44	−1.42 (0.30)	56	0.67
RV stroke volume, mL	36.78 (5.34)	44	39.47 (8.72)	56	0.06
RV stroke volume, mL/m^2.7^	20.16 (3.59)	44	20.38 (3.50)	56	0.77
RV ejection fraction, %	53.36 (5.49)	44	54.91 (5.63)	56	0.17

Data presented as mean (standard deviation) for parametric data or median (interquartile range) for nonparametric data or *N* (%).

### Cardiac computational atlas

**Figure 2:**
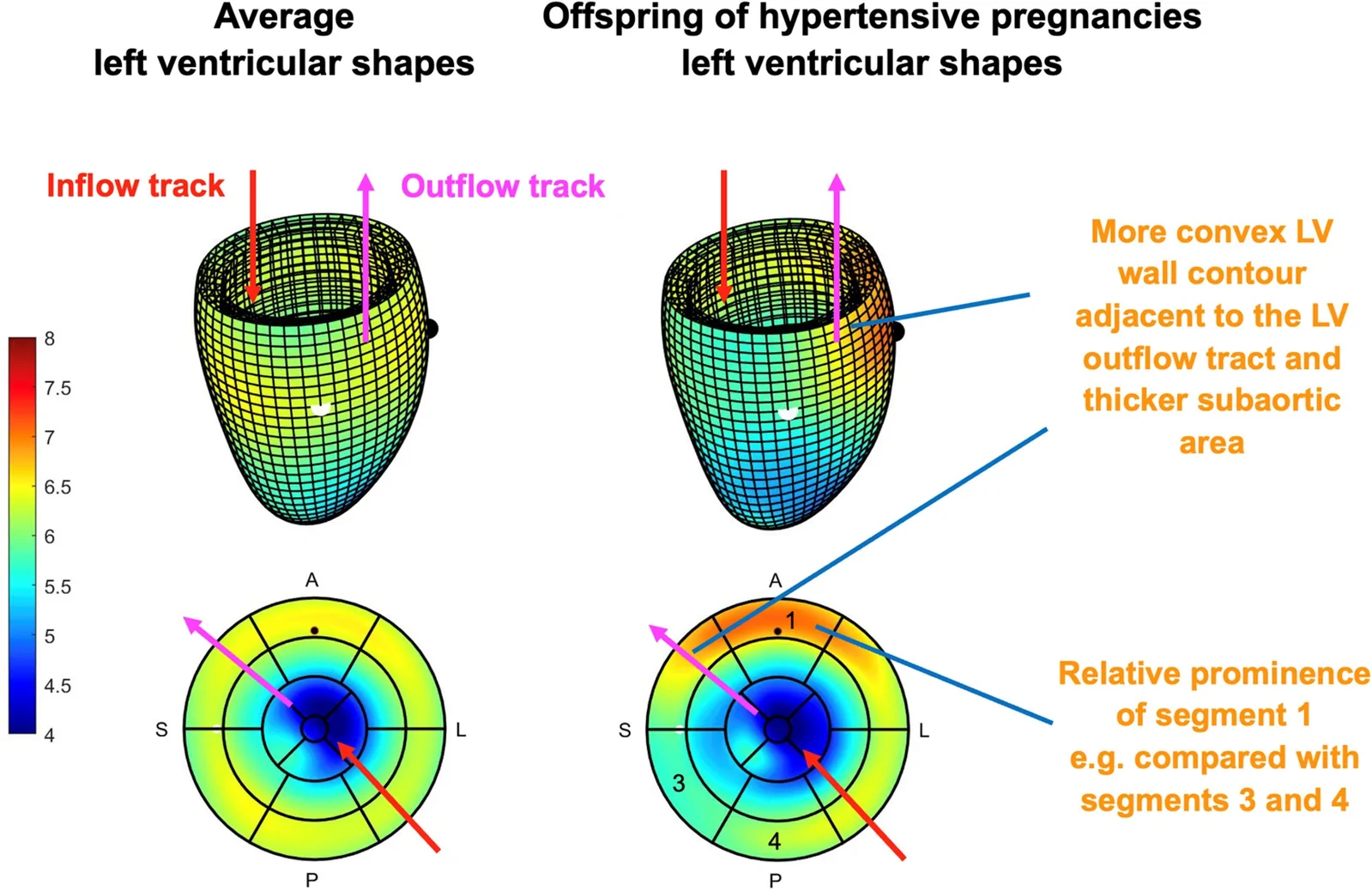
Cardiac shape analysis. Comparison of left ventricular (LV) morphology between offspring of hypertensive (HT) and normotensive (NT) pregnancies. Three-dimensional LV meshes and bullseye maps showing relative prominence of the basal anterior wall (Segment 1) and a more convex LV outflow tract (LVOT) in HT offspring. Colour scale indicates atlas-derived regional myocardial thickness (mm), illustrating coordinated regional morphological variations identified by the computational atlas rather than isolated conventional wall-thickness measurements (i.e. relative prominence of Segment 1 compared with posterior Segments 3 and 4). Black spheres mark the anterior aspect; white spheres indicate right ventricular region for orientation.

For the cardiac shape atlas, 42 scans from offspring of hypertensive pregnancies and 53 scans from normotensive pregnancies were included in the analysis. Five scans were excluded due to technical limitations. Cardiac shape modelling identified differences between groups (see Supplementary data online, *Figure S1*). *Figure 2* shows group differences across principal modes of variation. PCA identified Mode 18 as the key discriminator (*P* = 0.0144; area under the curve [AUC] = 0.635). Adding Mode 6 in linear discriminant analysis improved discrimination (*P* < 0.01; AUC=0.654), without altering the dominant morphological pattern. Across both approaches, two features distinguished offspring of hypertensive pregnancies from controls: (1) relative prominence of the basal anterior wall and (2) a more convex LVOT.

### Cardiac 4D flow CMR analysis

Of the 93 participants who underwent 4D Flow CMR acquisition, 67 met the predefined quality-control criteria for analysis. Baseline characteristics of included and excluded participants are summarized in Supplementary data online, *Table S2*. No meaningful differences were observed in demographic, perinatal, or clinical characteristics, including age, sex, exposure status, gestational age, birthweight, height, or blood pressure. Weight and body mass index were higher among excluded participants; however, these differences were small and not statistically significant (mean difference 1.28 kg, 95% CI −1.34 to 3.89; *P* = 0.33, and 0.70 kg/m^2^, 95% CI −0.23 to 1.63; *P* = 0.14, respectively). CMR–derived structural and functional parameters are shown in Supplementary data online, *Table S3*. No meaningful differences were observed between included and excluded participants.

**Figure 3:**
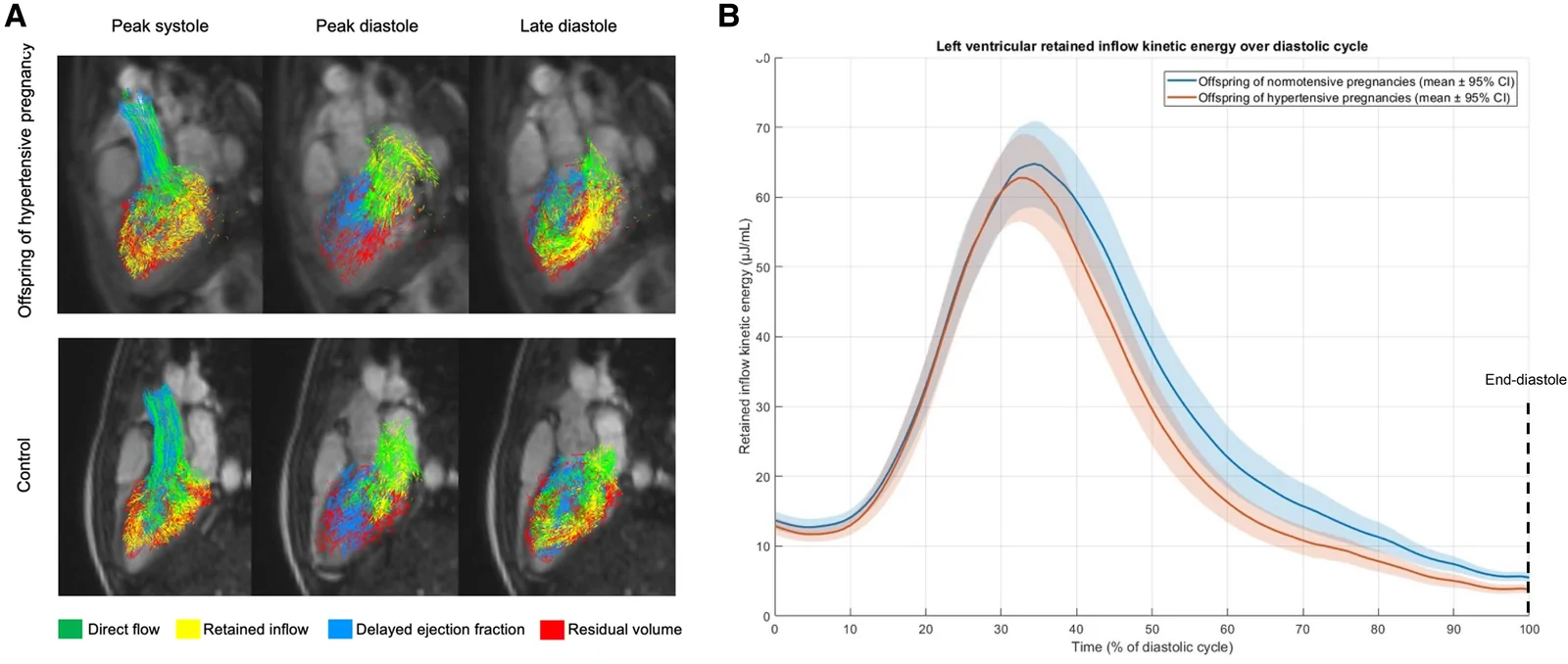
Left ventricular flow organization and retained inflow kinetic energy. **(***A***)** Intraventricular flow components visualized using 4D Flow CMR in three-chamber orientation at peak systole (left), peak diastole (middle), and late diastole (right) for offspring of hypertensive pregnancies (top row) and normotensive pregnancies (bottom row). Flow components are colour-coded as direct flow (green), retained inflow (yellow), delayed ejection flow (blue), and residual volume (red). Flow component volumes are comparable between groups by statistical volumetric analysis. **(***B***)** Time-resolved retained inflow kinetic energy (KE) indexed to component volume (μJ/mL) across the diastolic cycle, normalized to diastolic duration. Solid lines represent group means and shaded regions indicate 95% CI. Offspring of hypertensive pregnancies demonstrate lower retained inflow end-diastolic KE compared with controls (*P* < 0.001). The vertical dashed line marks the end-diastolic time point used for statistical comparison between groups.

Flow component analysis showed no differences between offspring of hypertensive and normotensive pregnancies in LV volumes of direct flow, retained inflow, delayed ejection flow, or residual volume. *Figure 3A* shows LV blood flow pathline visualizations.

KE indexed to component volume did not differ between groups at peak systole or peak diastole. At end-diastole, retained inflow KE indexed to retained inflow volume was lower in offspring of hypertensive pregnancies compared with controls (median 3.35 [IQR 3.03–4.80] vs. 5.70 [IQR 4.35–6.80] μJ/mL, *P* < 0.001) (*Table 3*). Exploratory subgroup analyses showed lower retained inflow end-diastolic KE in offspring exposed to PET and GH compared with controls (see Supplementary data online, *Table S4*). No differences were observed for KE in direct flow, delayed ejection flow, or residual volume components. *Figure 3B* shows time-resolved retained inflow KE profiles.

**Table 3:** Left intraventricular four-dimensional analysis of participants

Variables	Hypertensive pregnancies offspring	*N*	Normotensive pregnancies offspring	*N*	*P*-value	
Four-dimensional flow component					
Inflow, %	54.91 (6.33)	29	54.75 (5.31)	38	0.91
Outflow, %	51.33 (4.53)	29	50.24 (5.14)	38	0.37
Direct flow, %	32.97 (6.87)	29	32.18 (6.22)	38	0.63
Retained inflow, %	21.94 (2.99)	29	22.57 (2.75)	38	0.38
Delayed ejection flow, %	18.37 (3.28)	29	18.06 (3.05)	38	0.69
Residual volume, %	26.72 (4.59)	29	27.19 (5.20)	38	0.70
Four-dimensional flow end-diastolic kinetic energy (KE), indexed to the volume of the corresponding flow component					
Total end-diastolic KE, μJ/mL	4.44 (1.90)	29	4.86 (1.80)	38	0.36
Inflow end-diastolic KE, μJ/mL	5.37 (2.42)	29	6.14 (2.19)	38	0.18
Outflow end-diastolic KE, μJ/mL	6.06 (3.15)	29	6.04 (2.54)	38	0.98
Direct flow end-diastolic KE, μJ/mL	6.38 (3.29)	29	6.50 (2.64)	38	0.87
Retained inflow end-diastolic KE, μJ/mL	3.35 (3.03–4.80)	29	5.70 (4.35–6.80)	38	**<0**.001*
Delayed ejection flow end-diastolic KE, μJ/mL	5.49 (3.09)	29	5.23 (2.53)	38	0.71
Residual volume end-diastolic KE, μJ/mL	1.95 (0.85)	29	2.09 (0.95)	38	0.52

Data presented as mean (standard deviation) for parametric data or median (interquartile range) for non-parametric data or *N* (%).

*P < 0.0125, Bonferroni-corrected for multiple comparisons across four left ventricular flow components.

Multivariable linear regression analyses showed lower retained inflow end-diastolic KE in offspring exposed to hypertensive pregnancy. In models adjusted for sex and BMI, retained inflow end-diastolic KE was lower (B = −1.82, 95% CI −2.79 to −0.86; *P* = 0.0004). After additional adjustment for gestational age, results were similar (*B* = −1.73, 95% CI −2.68 to −0.77; *P* = 0.0006). Further adjustment for maternal BMI also showed a similar direction of effect (*B* = −1.65, 95% CI −2.80 to −0.50; *P* = 0.0056). In complete-case analyses restricted to term-born children (17 offspring of hypertensive pregnancies and 28 controls), the association remained similar in direction and statistical significance (*B* = −2.62, 95% CI −3.81 to −1.43; *P* < 0.001). No other KE measures differed.

## Discussion

### Principal findings

This study identifies subtle subclinical cardiac differences and altered intraventricular flow dynamics in children aged 6–9 years born to hypertensive pregnancies. The application of 4D Flow CMR in this age group enables detailed characterization of intraventricular flow patterns, providing novel insights into early cardiac phenotype. Despite preserved conventional CMR metrics, computational shape modelling identifies subtle regional LV differences, including relative prominence of the basal anterior wall and a more convex LVOT. These findings demonstrate modest discrimination between groups, indicating that the observed differences are evident at the group rather than individual level. The 4D Flow CMR analyses further demonstrate lower retained inflow end-diastolic KE. Together, these findings suggest subtle differences in cardiac phenotype detectable only with advanced imaging. As no individual PCA mode or KE metric was pre-specified as a primary endpoint, these findings require replication in independent cohorts.

### Conventional cardiac parameters: preserved global function

We observe no significant differences in the LV and RV volumes, ejection fraction, or global strain between groups. These findings are consistent with the Generation R cohort, which reported preserved LV measurements in mid-childhood among offspring of hypertensive pregnancies.^7^ However, we do not replicate their reported reduction in RV ejection fraction. While LV strain is also comparable in our cohort, a systematic review indicates that offspring of hypertensive pregnancies tend to exhibit reduced LV strain across developmental stages, albeit with variation in magnitude.^9^ The discrepancy between our findings and those from Generation R may reflect differences in cohort composition, including variation in the timing and type of hypertensive pregnancy. Limited sample size may also reduce power to detect subtle RV differences.

### Regional shape differences identified by cardiac shape analysis

Statistical shape modelling identifies subtle regional LV differences in offspring of hypertensive pregnancies, characterized by relative prominence of the basal anterior wall and a more convex LVOT. These regional morphological differences reflect coordinated three-dimensional variation identified by the computational atlas rather than isolated conventional wall-thickness measurements. The discriminative performance of the model is modest, indicating that the observed differences are evident at the group rather than individual level. Accordingly, these morphological differences are likely to lie within the spectrum of normal anatomical variation at the individual level and should be regarded as early subclinical features rather than markers with immediate clinical utility.

These shape analyses extend prior observations of early myocardial changes following hypertensive pregnancies. Vøgg et al. reported increased septal and posterior wall thickness in infants,^25^ while Aye et al. observed disproportionate myocardial hypertrophy in neonates exposed to maternal hypertension.^6^ Timpka et al. reported increased LV relative wall thickness in adolescents exposed to PET,^8^ suggesting concentric remodelling, and Lewandowski et al. demonstrated altered LV geometry and reduced longitudinal strain in young adults born preterm (mean gestational age 30.3 ± 2.5 weeks).^17^ In that study, reduced longitudinal peak systolic strain was also observed in young adults born preterm to PET, suggesting that PET may confer additional effects on cardiac phenotype beyond those attributable to prematurity alone.

In our cohort, offspring exposed to hypertensive pregnancies had lower gestational age. However, evidence from term-born infants^6^ and adolescents^8^ suggests that these observations are not fully explained by prematurity. The use of shape modelling extends these observations by providing spatial resolution, identifying specific anatomical regions involved in remodelling.

This study was not powered to assess sex-specific differences, although previous literature reports sex-related variation in cardiovascular outcomes following hypertensive pregnancies.^2^ In addition, genetic factors may contribute to myocardial geometry, as genome-wide association studies demonstrate overlap between loci influencing LV wall thickness and hypertension-related variants.^26^

### Altered diastolic flow characteristics revealed by 4D flow CMR

End-diastolic KE has been proposed as a marker of presystolic momentum and intraventricular flow organization.^23^ In our cohort, offspring of hypertensive pregnancies exhibit reduced retained inflow end-diastolic KE, despite similar KE across other flow components.

The physiological significance of retained inflow KE is less well defined than that of direct flow, which contributes more directly to early systolic ejection. Prior studies in dilated cardiomyopathy demonstrate deterioration of the retained inflow component,^22^ supporting its potential relevance to ventricular efficiency. In this context, the isolated reduction at end-diastole, in the presence of preserved KE at peak diastole, suggests reduced persistence of diastolic momentum rather than impaired early filling or atrial contribution.

Within the intraventricular energetics, KE can be redistributed to the resident blood, transiently stored as myocardial elastic energy, or dissipated as heat.^22^ The preservation of KE at peak diastole alongside reduced end-diastolic KE is therefore most consistent with increased late-diastolic energy loss, rather than a primary abnormality of filling pressure.

A proportion of participants were excluded from the 4D Flow analysis due to image quality or processing constraints. Baseline characteristics of included and excluded participants were comparable (see Supplementary data online, *Table S2*), with no significant differences in body size. Body size was accounted for in the regression models by adjustment for BMI, selected *a priori* based on previous studies^8,27^ as a parsimonious measure of overall body size and adiposity, particularly given the modest sample size and the need to limit model overfitting. Nevertheless, the reduced 4D Flow sample may limit the generalizability of the kinetic-energy findings to the broader cohort.

Consistent findings across models, including adjustment for gestational age and restriction to term-born children, suggest that the observed differences are not solely attributable to prematurity. As gestational age may lie on the causal pathway, these adjusted analyses should be interpreted as exploratory. Birthweight was also lower in offspring of hypertensive pregnancies and may reflect differences in foetal growth as well as gestational age. However, birthweight does not fully capture foetal growth, and residual confounding related to foetal growth restriction cannot be excluded. Given the observational design, the findings should be considered associative rather than causal. Persistence of findings after adjustment for gestational age and in the term-born sensitivity analysis suggests that the observed association is not solely explained by prematurity, although residual confounding related to prematurity cannot be completely excluded.

### Integrative interpretation: interplay of structure and function

The observed differences indicate subtle variation in cardiac structure and flow organization in offspring of hypertensive pregnancies. The regional LV shape differences identified by computational shape modelling, including relative prominence of the basal anterior wall and increased LVOT convexity, occur in anatomical regions adjacent to the aorto-mitral continuity, where retained inflow is typically positioned at end-diastole.^28^ However, the relationship between regional geometry and flow energetics was not directly assessed in the present study and therefore remains speculative. One possible explanation is that subtle differences in this fibrous, less compliant region could influence late-diastolic recoil and vortex organization, thereby affecting the persistence of diastolic momentum to end-diastole. The observed reduction in retained inflow KE may therefore be consistent with differences in late-diastolic flow organization. Large population-based studies have reported an increased risk of cardiovascular disease among offspring of hypertensive pregnancies.^29^ Although the present findings may provide mechanistic clues linking hypertensive pregnancy exposure with later cardiovascular risk, the present study was not designed to determine whether the observed imaging differences mediate or predict future cardiovascular outcomes.

In this study, conventional CMR measures and most 4D Flow parameters were similar between groups, with differences observed only in retained inflow KE at end-diastole. This isolated finding should be interpreted cautiously given the number of parameters assessed and the modest sample size, which may limit power to detect small differences. The absence of differences in strain and other flow components suggests that any variation in cardiac phenotype is subtle and not captured by conventional indices at this age.

Although retained inflow end-diastolic KE demonstrated the strongest between-group difference and remained consistent after multivariable adjustment, it emerged from a broader exploratory analysis of related KE measures. The finding was specific to retained inflow KE, as other end-diastolic flow parameters were not significantly different. This may reflect sensitivity to late diastolic flow organization and energy retention, although it may also represent a chance finding or an early, localized variation not yet reflected in broader flow patterns. Accordingly, the biological and clinical significance of this observation remains uncertain and requires confirmation in larger, independent cohorts.

### Study limitations

Several limitations should be noted. Recruitment was affected by the COVID-19 pandemic, requiring adjustment to visit age windows. The overall cohort size was modest and further reduced in the 4D Flow CMR subset due to motion artefact and acquisition challenges, which may have limited statistical power to detect smaller differences in other CMR parameters and may also limit the generalizability of the kinetic-energy findings. The relatively small number of offspring within each hypertensive pregnancy subtype limited statistical power for robust comparisons between PET and GH. Accordingly, these subgroup findings should be interpreted cautiously and require confirmation in larger cohorts. The cross-sectional design precludes causal inference, and longitudinal studies are required to determine the clinical relevance and persistence of the observed imaging findings.

## Conclusions

Children born to hypertensive pregnancies show subtle group-level differences in cardiac morphology and reduced retained inflow end-diastolic KE. These findings suggest early differences in cardiovascular phenotype, although their long-term clinical significance remains uncertain. Longitudinal studies are required to determine whether these differences persist or are associated with future cardiovascular outcomes.

## Supplementary Material

qyag145_Supplementary_Data

## Data Availability

Qualified researchers may request de-identified study data by submitting a proposal outlining research objectives, a statistical analysis plan, and data requirements. Requests are reviewed by an internal and external advisory committee, and approved data are shared under a data-sharing agreement. Requests should be directed to the corresponding author.

## Lead author biography

Adam James Lewandowski is an Associate Professor of Cardiovascular Science at the Nuffield Department of Population Health, University of Oxford, and Deputy Chief Scientist for UK Biobank. He completed his undergraduate studies in biological sciences at the University of Guelph, Canada, followed by a Master’s degree at the University of Cambridge in Healthcare Data Informatics and a DPhil in Cardiovascular Medicine at the University of Oxford funded by a Commonwealth Scholarship. After completing his DPhil, he undertook postdoctoral research at the University of Oxford and was then awarded a British Heart Foundation Intermediate Research Fellowship. His research has primarily focused on multimodality cardiovascular phenotyping to understand the early-life origins of cardiovascular disease, particularly the long-term cardiovascular consequences of preterm birth and hypertensive pregnancy. At UK Biobank, Adam helps shape the scientific strategy for major cohort enhancements, including large-scale imaging and repeat assessment initiatives.
