# Supplementary material for: Cardiac morphology and flow dynamics in children born to hypertensive pregnancies
Source: Eur Heart J Imaging Methods Pract. 2026 Aug 20;4(3):qyag145. doi: 10.1093/ehjimp/qyag145 (PMC13543680; doi:10.1093/ehjimp/qyag145)
Supplement: Supplementary file 1 [file qyag145_supplementary_data.docx]

**Supplementary Materials**

Supplementary Figure S1. Cardiac shape analysis.

This figure compares left ventricular (LV) morphology between offspring of hypertensive pregnancies (HT) and normotensive pregnancies (NT). Panel A: p-values from t-tests across principal component analysis (PCA) modes, with Mode 18 significantly differentiating groups (p = 0.0144). Panel B: Deviance test and t-test histograms demonstrating between-group variation in PCA score distributions (deviance test p = 0.0041; t-test p = 0.0057). The accompanying scatter plot displays Mode 6 vs. Mode 18 with the linear discriminant boundary illustrating group separation. The adjacent bar chart shows PCA mode weights for Modes 6 and 18 contributing to the linear discriminant. PCA mode selection and testing were exploratory and data-driven.

Supplementary Table S1. Criteria of adequate four-dimensional flow image quality for intraventricular flow analysis

No significant artefact from visual inspection and full cardiac cycle coverage

AND

Ventricular stroke volume from 4D Flow CMR analysis >80% of stroke volume from volumetrics

AND

Ventricular inflow and outflow discrepancy* from 4D Flow CMR analysis <15%

AND

Ventricular junk proportion <25%

*Ventricular inflow and outflow discrepancy percentage was calculated as the ratio of inflow-outflow difference to the average of inflow and outflow

Supplementary Table S2. Key baseline characteristics of participants included in and excluded from the 4D flow analysis.

| Variables | Included participants | N | Excluded participants | N | P value |
| --- | --- | --- | --- | --- | --- |
| Age, years | 7.48 (0.94) | 67 | 7.38 (1.03) | 33 | 0.63 |
| Male | 39 (58.2%) | 67 | 18 (54.5%) | 33 | 0.89 |
| Maternal hypertension during pregnancy | 29 (43.3%) | 67 | 15 (45.5%) | 33 | >0.99 |
| Preeclampsia | 20 (29.9%) | 67 | 11 (33.3%) | 33 | 0.94 |
| Gestational hypertension | 9 (13.4%) | 67 | 4 (12.1%) | 33 |  |
| Gestational diabetes mellitus | 4 (6.2%) | 64 | 2 (7.4%) | 27 | >0.99 |
| Gestational age, weeks | 37.66 (3.37) | 67 | 37.65 (3.45) | 33 | >0.99 |
| Prematurity | 22 (32.8%) | 67 | 10 (30.3%) | 33 | 0.98 |
| Antenatal steroids | 15 (23.4%) | 64 | 8 (29.6%) | 27 | 0.72 |
| Birth weight, g | 2980.45 (948.51) | 67 | 2982.85 (873.23) | 33 | 0.99 |
| Weight, kg | 26.44 (5.92) | 67 | 27.72 (6.27) | 33 | 0.32 |
| Height, cm | 126.51 (7.90) | 67 | 126.90 (9.79) | 33 | 0.83 |
| Body mass index, kg/m^2^ | 16.34 (2.08) | 67 | 17.04 (2.24) | 33 | 0.13 |
| Systolic blood pressure, mmHg | 104.30 (7.98) | 67 | 104.18 (8.21) | 33 | 0.95 |
| Diastolic blood pressure, mmHg | 59.48 (5.20) | 67 | 59.33 (4.58) | 33 | 0.89 |

Values are presented as mean (standard deviation) or N (%). N denotes the number of non-missing observations per variable. Included participants (N=67) had analysable 4D Flow CMR data. Excluded participants (N=33) comprised those with unavailable 4D Flow data (N=7) and those excluded due to insufficient image quality or processing constraints (N=26).

Supplementary Table S3. Cardiac structure and function of participants included in and excluded from the 4D flow analysis.

|  | Variables | Included participants | N | Excluded participants | N | P value |
| --- | --- | --- | --- | --- | --- | --- |
| CMR ventricular structural parameters | |  |  |  |  |  |
|  | LV mass, g | 45.14 (9.92) | 67 | 44.39 (8.54) | 33 | 0.71 |
|  | LV mass index, g/m^2.7^ | 23.85 (3.82) | 67 | 23.46 (4.23) | 33 | 0.64 |
|  | LV end-diastolic volume, mL | 67.97 (12.54) | 67 | 67.81 (11.69) | 33 | 0.95 |
|  | LV end-diastolic volume, mL/m^2.7^ | 35.93 (4.66) | 67 | 35.86 (5.87) | 33 | 0.95 |
|  | LV mass/end-diastolic volume, g/mL | 0.67 (0.08) | 67 | 0.66 (0.07) | 33 | 0.57 |
|  | LV relative wall thickness | 0.28 (0.05) | 67 | 0.27 (0.05) | 33 | 0.25 |
|  | LV length at end-diastole, mm | 70.42 (5.78) | 64 | 70.36 (6.01) | 33 | 0.96 |
|  | RV end-diastolic volume, mL | 70.85 (13.57) | 67 | 70.96 (13.28) | 33 | 0.97 |
|  | RV end-diastolic volume, mL/m^2.7^ | 37.40 (4.69) | 67 | 37.48 (6.53) | 33 | 0.95 |
|  | RV base diameter at end-diastole, mm | 36.21 (3.85) | 67 | 36.17 (4.76) | 33 | 0.96 |
|  | RV mid diameter at end-diastole, mm | 25.54 (3.66) | 64 | 25.78 (4.47) | 33 | 0.77 |
|  | RV length at end-diastole, mm | 63.45 (5.75) | 67 | 63.61 (6.79) | 33 | 0.90 |
| CMR ventricular functional parameters | |  |  |  |  |  |
|  | LV stroke volume, mL | 40.43 (7.58) | 67 | 40.34 (6.35) | 33 | 0.95 |
|  | LV stroke volume, mL/m^2.7^ | 21.39 (2.99) | 67 | 21.44 (3.89) | 33 | 0.94 |
|  | LV ejection fraction, % | 59.61 (4.15) | 67 | 59.79 (4.71) | 33 | 0.84 |
|  | LV longitudinal systolic strain, % | -18.56 (2.10) | 67 | -18.55 (1.93) | 33 | 0.98 |
|  | LV longitudinal systolic strain rate, s^-1^ | -1.04 (0.15) | 67 | -1.04 (0.16) | 33 | 0.98 |
|  | LV basal circumferential systolic strain, % | -18.74 (2.04) | 67 | -18.86 (2.05) | 33 | 0.78 |
|  | LV basal circumferential systolic strain rate, s^-1^ | -1.14 (0.15) | 67 | -1.15 (0.16) | 33 | 0.82 |
|  | LV mid circumferential systolic strain, % | -18.14 (1.93) | 67 | -18.19 (2.21) | 33 | 0.90 |
|  | LV mid circumferential systolic strain rate, s^-1^ | -1.12 (0.12) | 67 | -1.09 (0.12) | 33 | 0.34 |
|  | LV apical circumferential systolic strain, % | -20.44 (3.10) | 67 | -20.18 (3.19) | 33 | 0.69 |
|  | LV apical circumferential systolic strain rate, s^-1^ | -1.40 (0.23) | 67 | -1.49 (0.30) | 33 | 0.13 |
|  | RV stroke volume, mL | 38.24 (7.91) | 67 | 38.38 (6.77) | 33 | 0.93 |
|  | RV stroke volume, mL/m^2.7^ | 20.21 (3.19) | 67 | 20.44 (4.17) | 33 | 0.75 |
|  | RV ejection fraction, % | 54.10 (5.68) | 67 | 54.48 (5.49) | 33 | 0.75 |

Values are presented as mean (standard deviation). N denotes the number of non-missing observations per variable. Included participants (N=67) had analysable 4D Flow CMR data. Excluded participants (N=33) comprised those with unavailable 4D Flow data (N=7) and those excluded due to insufficient image quality or processing constraints (N=26).

Supplementary Table S4. Cardiac four-dimensional flow analysis of the offspring according to the type of maternal hypertensive pregnancies. Both offspring of preeclampsia and gestational hypertension exhibited reduced retained inflow kinetic energy.

|  | Variables | Preeclamptic offspring | N | Gestational hypertension offspring | N |
| --- | --- | --- | --- | --- | --- |
| Four-dimensional flow component | |  |  |  |  |
|  | Inflow, % | 54.59 (6.14) | 20 | 55.60 (7.08) | 9 |
|  | Outflow, % | 51.10 (3.85) | 20 | 51.86 (6.01) | 9 |
|  | Direct flow, % | 32.46 (6.39) | 20 | 34.09 (8.12) | 9 |
|  | Retained inflow, % | 22.13 (2.63) | 20 | 21.51 (3.83) | 9 |
|  | Delayed ejection flow, % | 18.64 (3.35) | 20 | 17.77 (3.21) | 9 |
|  | Residual volume, % | 26.77 (4.06) | 20 | 26.63 (5.89) | 9 |
| Four-dimensional flow end-diastolic kinetic energy (KE), indexed to the volume of the corresponding flow component | |  |  |  |  |
|  | Total end-diastolic KE, μJ/mL | 4.65 (2.04) | 20 | 3.97 (1.55) | 9 |
|  | Inflow end-diastolic KE, μJ/mL | 5.61 (2.52) | 20 | 4.82 (2.23) | 9 |
|  | Outflow end-diastolic KE, μJ/mL | 6.33 (3.39) | 20 | 5.47 (2.64) | 9 |
|  | Direct flow end-diastolic KE, μJ/mL | 6.68 (3.47) | 20 | 5.69 (2.90) | 9 |
|  | Retained inflow end-diastolic KE, μJ/mL | 4.03 (3.00-5.10) | 20 | 3.21 (3.06-3.67) | 9 |
|  | Delayed ejection flow end-diastolic KE, μJ/mL | 5.76 (3.38) | 20 | 4.89 (2.40) | 9 |
|  | Residual volume end-diastolic KE, μJ/mL | 2.05 (0.90) | 20 | 1.73 (0.72) | 9 |

KE = kinetic energy.

Data are presented as mean (standard deviation) for normally distributed variables, median (interquartile range) for non-normally distributed variables, or N (%). N denotes the number of non-missing observations per variable.
